# Internet Patient Records: new techniques

**DOI:** 10.2196/jmir.3.1.e8

**Published:** 2001-03-17

**Authors:** Gavin Brelstaff, Sascha Moehrs, Paolo Anedda, Massimiliano Tuveri, Gianluigi Zanetti

**Affiliations:** ^1^CRS4, BioMedical AreaSardiniaItaly

**Keywords:** Electronic Medical Record, Medical Information Systems, Internet, Java, JavaScript, XML, XSL, Rapid Prototyping, Elicitation Methods

## Abstract

**Background:**

The ease by which the Internet is able to distribute information to geographically-distant users on a wide variety of computers makes it an obvious candidate for a technological solution for electronic patient record systems. Indeed, second-generation Internet technologies such as the ones described in this article - XML (eXtensible Markup Language), XSL (eXtensible Style Language), DOM (Document Object Model), CSS (Cascading Style Sheet), JavaScript, and JavaBeans - may significantly reduce the complexity of the development of distributed healthcare systems.

**Objective:**

The demonstration of an experimental Electronic Patient Record (EPR) system built from those technologies that can support viewing of medical imaging exams and graphically-rich clinical reporting tools, while conforming to the newly emerging XML standard for digital documents. In particular, we aim to promote rapid prototyping of new reports by clinical specialists.

**Methods:**

We have built a prototype EPR client, InfoDOM, that runs in both the popular web browsers. In this second version it receives each EPR as an XML record served via the secure SSL (Secure Socket Layer) protocol. JavaBean software components manipulate the XML to store it and then to transform it into a variety of useful clinical views. First a web page summary for the patient is produced. From that web page other JavaBeans can be launched. In particular, we have developed a medical imaging exam Viewer and a clinical Reporter bean parameterized appropriately for the particular patient and exam in question. Both present particular views of the XML data. The Viewer reads image sequences from a patient-specified network URL on a PACS (Picture Archiving and Communications System) server and presents them in a user-controllable animated sequence, while the Reporter provides a configurable anatomical map of the site of the pathology, from which individual "reportlets" can be launched. The specification of these reportlets is achieved using standard HTML forms and thus may conceivably be authored by clinical specialists. A generic JavaScript library has been written that allows the seamless incorporation of such contributions into the InfoDOM client. In conjunction with another JavaBean, that library renders graphically-enhanced reporting tools that read and write content to and from the XML data-structure, ready for resubmission to the EPR server.

**Results:**

We demonstrate the InfoDOM experimental EPR system that is currently being adapted for test-bed use in three hospitals in Cagliari, Italy. For this we are working with specialists in neurology, radiology, and epilepsy.

**Conclusions:**

Early indications are that the rapid prototyping of reports afforded by our EPR system can assist communication between clinical specialists and our system developers. We are now experimenting with new technologies that may provide services to the kind of XML EPR client described here.

## Introduction

Many European countries aim to introduce Electronic Patient Records (EPRs) as part of the modernization of their public health services. Indeed, the UK NHS (United Kingdom National Health Service) Executive [1] reported that:

2.7 The arguments for a move towards an electronic record are compelling. Such records are more likely to be legible, accurate, safe, secure, and available when required, and they can be more readily and rapidly retrieved and communicated. They better integrate the latest information about a patient's care, for example from different "departmental" clinical systems in a hospital. In addition, they can be more readily analyzed for audit, research and quality assurance purposes.

Two key benefits that they list are the *integration of care* between general practitioners (GPs) and hospitals, and *improving efficiency* by reducing the time spent by health professionals collecting existing data. Hospitals are often critical about the inadequate and incomplete information to support referrals. GPs in turn consistently complain about the quality and timeliness of test results and information following outpatient or inpatient care. In sum, the quality of clinical communication between hospitals and GPs presents a fundamental challenge to the quality and safety of patient care.

The ease by which the Internet is able to distribute information to geographically-distant users on a wide variety of computers makes it an obvious candidate for a technological solution for an electronic patient record system. Indeed, our previous project, WMED [2] illustrated how first-generation Internet technology could provide a useful infrastructure for secure EPR retrieval.

Yet, providing a nationwide healthcare information infrastructure based on Internet protocols, such as the UK's NHSnet, addresses only part of the electronic solution [3]. Confidentiality of communications and data storage must be guaranteed [4,5,6]. Furthermore, interoperable clinical applications should also be provided. Ensuring effective interoperation between heterogeneous applications generally requires adopting standard protocols and/or data formats. In the healthcare domain, such standards are usually issued by committee: HL7 (Health Level Seven), EDI/EDIFACT (Electronic Data Interchange/Electronic Data Interchange For Administration, Commerce and Transport), DICOM (Digital Imaging and Communications in Medicine), CEN (European Normalization Committee) [7], or imposed by proprietary software vendors. This has tended to inhibit the development of clinical applications that require network transactions. However, this may now change with the advent of a global open-standard for representing documents, XML (eXtensible Markup Language) [8]. In this article, we harness XML along with other second-generation Internet technologies to demonstrate new techniques for building highly interactive EPR web-clients.

XML, with its inherent facility to impose application-specific constraints on the hierarchy and data content of documents, appears well suited for specifying EPRs. XML is, in fact, a distilled version of SGML (Standard Generalized Markup Language), a more general standard by which rather more complex constraints can be applied. SGML's great complexity meant that it was not widely adopted for healthcare applications, a notable exception being a medical markup language project in Japan [9]. XML's greater accessibility has subsequently led to the wide availability of tools for its development. Indeed, major standards committees are now incorporating XML within their existing standards (eg, Structured Reporting in DICOM [10], Patient Record Architecture in HL7 [11], and the XML-EDI initiative). Independent pilot projects, like the Scottish Immediate Discharge Document system [12], are also deploying XML.

Containment of system complexity has been a primary motivation throughout the evolution of second-generation Internet technologies. For example, XML specifies the content of a document independently of how it should be presented and independently of the logic associated with its interactions. This can help simplify the design of an EPR system. Document content delivered as XML can be presented on the web through the application of stylesheets that conform to well-defined open standards: XSL (eXtensible Style Language) [13], to lay out the format; and CSS (Cascading Style Sheets) [14], for page-color and typographical styling.

Specifying the ways users can interact with the document presented to them (eg, to update or add to it) is intrinsically coupled to the way the content itself has been specified. Here, two different approaches have emerged to keep this specification reasonably simple. One has been to embed so-called event handling in the XML technology (as we largely adopt here), while the other has been to follow a comprehensive object-oriented (OO) paradigm in which data-content and the methods to manipulate it both become wrapped into true program objects. In theory an object can be strongly decoupled from other objects within a system and consequently overall complexity ought to be kept low. Ideally a web of objects [15] would communicate to achieve a joint purpose (eg, the maintenance of the EPR). In practice, complexity inevitably re-emerges when programs distribute their constituent objects over different nodes of a heterogeneous network even when standard interfacing and protocols - eg, CORBA/IDL/IIOP (Common Object Request Broker Architecture/Interface Definition Language/Internet Inter-ORB Protocol), Java/RMI (Java/Remote Method Invocation), or MS/DCOM (Microsoft/Distributed Component Object Model) - are adopted. In particular, tolerating temporary network failure can have significant impact on the complexity of object-to-object collaboration. Nevertheless, distributed object-oriented approaches do exist in the healthcare domain, notably the CORBAmed initiative and the HANSA (Healthcare Advanced Network System Architecture) system [16].

The DOM (Document Object Model) [17] provides the standard way to interact with XML documents. Web browsers have contained a primitive DOM (retrospectively named level-0) that applied to HTML form and link elements. Specifying user interaction on a web page was achieved by providing scripts, written in JavaScript, to specify how events such as clicking and selecting items were to be handled. The latest versions of the Internet Explorer and Netscape (Mozilla) web-browsers now embed more comprehensive, standards-based DOMs that are automatically populated whenever they receive an XML document. In effect, a mini-database (eg, a tree hierarchy representing patient data) resides for the duration of the web page on the client computer ready to be presented to, and manipulated by, the clinician. Again, the event handlers can be scripted to achieve programmatic interactivity. However, a barrier to principled approach here is the lack of explicit data-typing in XML delivered to the DOM. Until recently a Document Type Declaration (DTD) was all that was available to specify the rules governing document content, and those rules treated all data as character strings. However, this deficiency may soon be overcome by an emerging standard XML-Schema [18].

In anticipation of the arrival of web browsers that furnish access to a data-typed DOM, we have built a system that emulates some of the effects in today's (version 4 and 5) browsers. To do this we employ a combination of JavaScript and Java applets. Java allows us to incorporate a public-domain DOM/XML parser and XSL transformation methods [19]. It also provides a sophisticated OO programming environment when the need arises (eg, to implement clinical image viewing or decision support). Java applets have the benefits of being portable, transparently downloadable, and able to interface with JavaScript event handlers on the web page. Furthermore, by adopting the JavaBean [20] methodology each of our applets is made into an independent software component. This helps keep complexity under control, as does formalizing inter-component communication to use the Java InfoBus [21] class-library. Communication between components running in the same Java Virtual Machine is supported. We do not ask our JavaBeans to communicate directly with others across the network, but instead rely on the browser's innate asynchronous connectivity to download/upload resources according to standard Internet protocols. That way our system can remain fairly tolerant to transitory network disconnectivity.

## Methods

**Figure 1:**
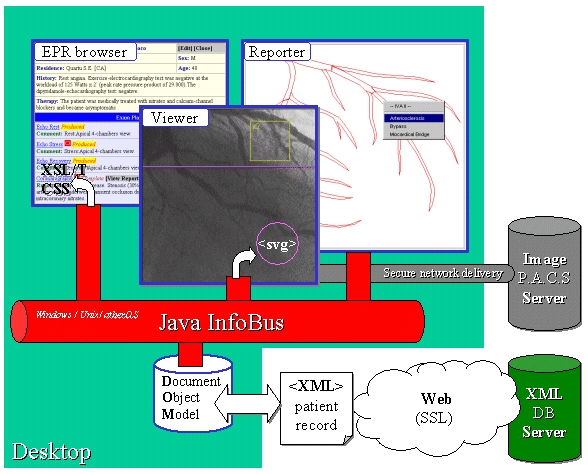
The electronic patient record is delivered over the Web via secure SSL as an XML document. On the client it is stored in the Document Object Model and thus its contents become available for presentation in a variety of ways. The basic patient details can be viewed as an interactive web page (by the application of XSL and CSS stylesheets). From the EPR Browser other views of the same record can be launched; the image Viewer and the clinical Reporter JavaBeans are shown. These tools have been used to modify the record so that it can be uploaded back to the server. Communication among JavaBeans is mediated by the InfoBus, while the launch of new windows is achieved using JavaScript libraries

We have built a prototype EPR client, named InfoDOM, that runs in both the popular web browsers: Internet Explorer 4 & 5, and Netscape 4. Figure 1 illustrates the basic mechanisms involved in this system. Here, version 2 of our prototype is described; we presented the first version at the PA Java 2000 conference [22]. The process begins by an IO (Input/Output) Manager bean (not shown) requesting an XML record over the web from the EPR server. For this we have used the Apache web server running SSL (Secure Socket Layer). The downloaded document is then stored in the client-side DOM.

**Figure 2:**
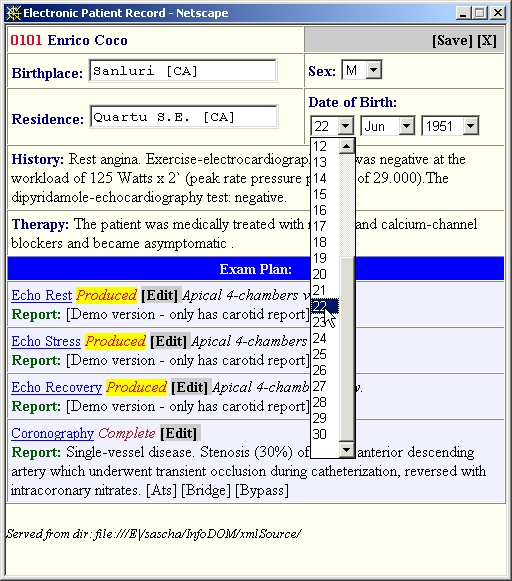
Parts of the XML patient record are rendered as a web page, the EPR Browser, in a pop-up browser window (shown). In addition to text summary items such as History, Therapy, and Report, this page contains several interactive items: form fields, such as Date of Birth (shown here as it is being modified); links to viewable imaging exams that are shown as underlined (eg, Coronography); and links to the graphical Reporter tool, to view and edit a report of the exam in question

An XSL file is also downloaded and then applied to the DOM content by the XSL Processor bean (not shown) to produce HTML. A pop-up web page (the EPR Browser) defined by that HTML is then generated. From that page users can begin to work on the patient data, simply by clicking on the appropriate contextual links. For example, users may choose to modify the textual data as shown in Figure 2, or else they may launch further pop-up windows containing the Image Viewer or Reporter beans parameterized appropriately for the particular patient and exam in question.

**Figure 3:**
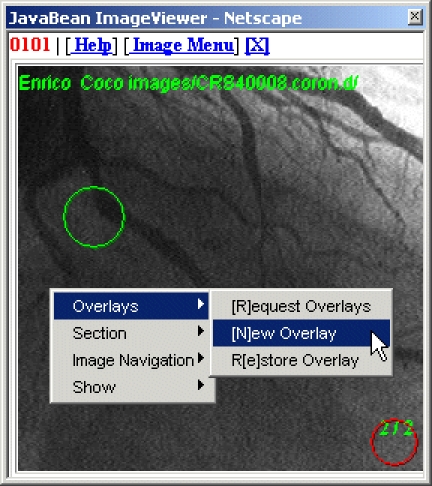
The JavaBean Image Viewer is capable of: showing an animated sequence; creating and modifying annotated graphical overlays; and then saving them in Scalable Vector Graphic format inside the DOM. In addition, parts of the image can be magnified and gray-level sections graphed (not shown)

The Image Viewer reads image sequences from a patient-specified network URL on a PACS (Picture Archiving and Communications System) server and presents them in a user-controllable animated sequence. The animation can be halted to examine any particular image frame in detail. For example a 2-, 3-, or 4-times magnifier window can be made to track the mouse cursor. Image regions of interest can be annotated using line graphic overlays as illustrated in Figure 3. These overlays are read and written to the DOM and form parts of the report section. We leave the original image data as a read-only resource. This is because synchronizing updates to large image sequences across networked clients would inevitably be prone to delays and would require complex back-end integration with the PACS servers. The Scalable Vector Graphics (SVG) standard [23] is used to represent the overlays. As SVG is simply a dialect of XML, it is possible to store annotations in the DOM and transport them as XML fields. Our prototype client currently reads GIF images from a web directory; however it is not difficult to extend the system to read images from a PACS. We have carried out preliminary tests [24,25] using a DICOM 3 compliant PACS [26]. As such, we are in line with other researchers [27,28].

**Figure 4:**
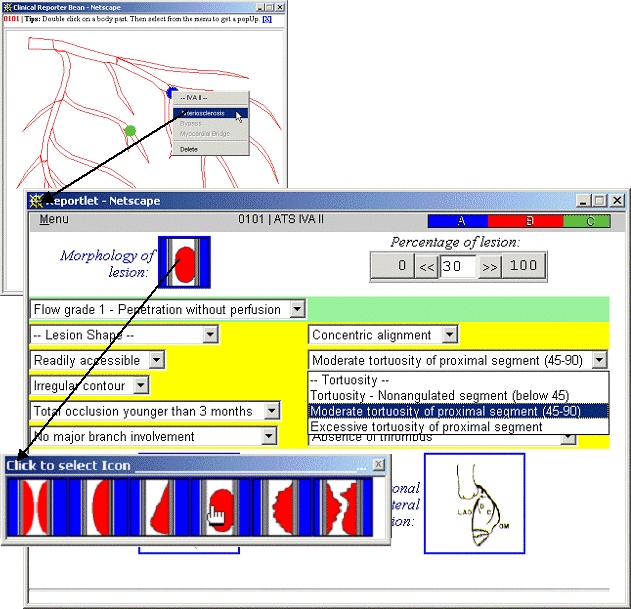
The Reporter Bean (top-left) is launched from the EPR Browser. Each report is configured to the particular exam in question, here the left coronary artery. The user clicks on the resultant image map (SVG encoded) to initiate a reportlet page from a menu of context-sensitive reportlets. Here, the user chose to report on Arteriosclerosis for the vessel labeled IVA II. A reportlet (bottom) for Arteriosclerosis then appears and allows the medics to graphically specify the clinical signs appropriate for their patient. As is shown, the interface is constructed from sliders, pull-down text menus and pop-up image menus; there is no way to hand type information. The colored part of the menu-bar is a decision support aid that dynamically indicates the type of lesion, based on the evidence accumulated in the report. In this case the simple voting heuristic implemented indicates that a type B lesion is the most likely interpretation. The next figure illustrates the mechanism underlying each reportlet

Clicking on the [Edit] link in theEPR Browser launches a context-specific Reporter tool. Figure 4 (top) illustrates the configuration for reporting on coronary arteries. The design follows an anatomical image map paradigm. The image map structure is read as SVG and its context-sensitive pop-up menu is specified in XML. For example the figure shows the IVA-II artery and a pop-up menu that can launch three different types of report: Arteriosclerosis, Bypass, or Myocardial Bridge. Selecting a menu item launches a pop-up reportlet window; the Arteriosclerosis reportlet window is illustrated in Figure 4 (bottom).

The Arteriosclerosis reportlet allows the medics to graphically specify the clinical signs appropriate for their patient's coronary vessel IVA II. The interface is constructed from sliders, pull-down text menus, and pop-up image menus, all of which are implemented via JavaScript library calls. Wherever feasible the user is presented with a limited range of items or numerical values to select from, thus minimizing difficulties in handling hand-typed text. The menu-bar at the top of every reportlet is, in fact, an embedded JavaBean that serves two functions: the reading and writing of data content to and from the DOM via the InfoBus; and the provision (when appropriate) of decision support information. For example, in Figure 4 the colored part on the right of the menu-bar adjusts dynamically to suggest the type of lesion, based on the evidence accumulated in the report. We have initially implemented a simple voting system. The medic may author each form element in such a way as to specify how its possible values can influence the probable diagnosis. For example, in the figure the choice being made, "Moderate tortuosity of proximal segment," will produce one vote for the diagnosis of a type B lesion.

An early version of this Arteriosclerosis tool [29] was included in the WMED system, but there it was not integrated into the EPR content. With InfoDOM the data content is now written and read from the DOM and transferred as XML. The current prototype provides five other such reportlets. All graphical assets, such as image-menus and SVG maps, follow a web directory style hierarchical organization and so are instantly transportable between websites or to hard disk.

**Figure 5:**
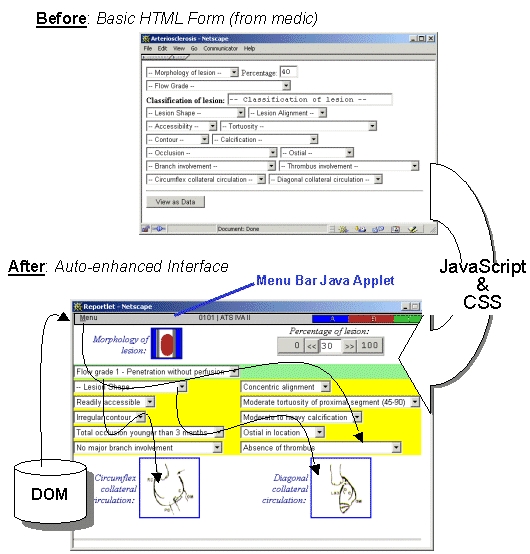
The enhancement mechanism for reportlets: a reportlet HTML form (top) prototyped by the medic is loaded. Next, a JavaScript routine examines the contents of each element of the form, to identify the elements marked up for enhancement (eg, icon-menus and slider-bars). This generates a new page (bottom) in which elements are tabulated and colored according to a CSS stylesheet. The menu-bar inserted at the top of the new page is, in fact, a Java Applet that communicates with the DOM to read (and later write) the stored contents of the reportlet. Thus, once the new page has loaded, the menu-bar automatically populates the form elements (including the enhanced elements) of the page. Key to each step of this mechanism is the names of the elements in the original form. Indeed, those names identify the content of the XML fields that will be used in the EPR

Version 2 of InfoDOM has rationalized and generalized the implementation of reportlets. JavaScript libraries have been written to generate arbitrarily specified reportlets. This approach provides an improvement in speed, stability, and configurability over our previous version that employed the Java AWT (Abstract Window Toolkit) to do this task. In particular, the gamut of interactions and choices available within a given reportlet is now specified by writing individual HTML forms following a small set of simple rules. These forms get automatically enhanced when placed inside the InfoDOM environment. Away from the InfoDOM environment they can be created using any HTML editor and viewed (unenhanced) in any browser. This makes it possible for a specialist in a particular field to specify a detailed set of reporting parameters and constraints on the parameters, without the need to interact with our system developers. The browser on a specialist's desktop is sufficient for the specialist to explore the interactive functionality of the part of the system that is being designed. Once the specialist is satisfied with the content of the HTML form, it is incorporated into the InfoDOM system (a) by selecting a CSS style for its element, and (b) by ensuring it calls the appropriate JavaScript library. Figure 5 illustrates the mechanisms involved in generating an enhanced form:

1. The reportlet HTML form coded by the medic is loaded as a web page.
2. As soon as the page is loaded, a JavaScript routine examines the contents of each element of the HTML form (ie, it interrogates the level-0 DOM).
3. The script then identifies those elements that have been marked up for enhancement - eg,
  - A select box to transform into an icon menu (eg, the small pop-up in Figure 4 (bottom) marked "Click to select Icon"). Here, a set of GIF-icons that correspond to each select option has been prespecified by the medic.
  - A numerical text field to transform into a slider-bar (eg, the field in Figure 4 (bottom) marked "Percentage of lesion"). Here, a fixed range of values has been requested.
  - A date text field to transform into a day-month-year 3-way selection combo (eg, the field shown in Figure 2). Here, the combo is dynamically constrained by an algorithm to allow only legal dates (eg, leap days).
4. The script then generates a new page that re-represents each form element, substituting those that it can with enhanced versions and spatially formatting them in cells of a regular table.
5. Colors are assigned to each table cell according to the name of its element, using a custom-built CSS stylesheet. That name is the one which the medic specified in the original form using the standard attribute assignment: NAME="yourNameInHere".
6. That name, more importantly, identifies the DOM data-item to which that form element is to read and write its contents. In fact, each element in the new page can be updated with the correspondingly named DOM data-item as soon that page has been loaded into a browser window. A JavaBean, that manifests as the menu-bar of that page, carries out this task by communicating with the DOM and appropriately adjusting the value of the elements of the form in the enhanced page.

To achieve a stable synchronization of the above operations, we use a multi-frame web page (a frameset) so that the original form loads into an unseen frame, then the enhanced page loads into the main frame, and finally the JavaBean menu-bar loads into its own strip-like frame at the top.

The XML specification of each clinical section of the EPR is *produced by example* when one follows the above methodology. Although this may be rather unconventional, it does promote a rapid prototyping style of development. In fact, each particular reportlet specifies a particular branch of the XML tree structure. Part of the specialist's skill is to comprehensively specify the options needed to make a particular report, while another part is to know where to cut each branch so as to best differentiate between distinct pathologies. The spatial image-map interface of the Reporter component can help guide the latter decision. In any case, once a reporting regime has been established by example it is a minor task for an XML developer to convert the medic's original form into a formal XML specification.

## Results

The experimental system illustrated above is currently being adapted for use in three hospitals in Cagliari, Sardinia. Each site constitutes a test-bed system [30] in which medics are participating as both users (that provide feedback) and designers (that provide clinical reports), according to their particular medical specialties. The test-beds include:

1. The support for clinical reporting between radiologists and neurologists. Here, DICOM-compliant MRI (Magnetic Resonance Imaging) and CT (Computed Tomography) devices are used by radiologists to acquire imaging exams of the brain. These are stored on a PACS archive and thus made available to networked neurologists in different parts of the same hospital. The electronic patient record acts, here, as a skeleton upon which to collate information on individual exams for each participating patient.
2. The support for reporting visits by outpatients to epilepsy centers. Here, we aggregate details gathered over visits made at different times. These details include seizure descriptions, EEG and MRI exam reports, and responses to drug therapies. Here, the electronic patient record acts as a dynamic repository that accumulates over time. By visualizing how the patient's conditions evolve over time, the medic ought to be better able to make diagnoses and more accurately prescribe treatments.
3. The support for reporting visits by patients to an oncology hospital. Here, the time evolution of the size of tumors in response to various cancer treatments is to be visualized

It is during the design phase that our system's rapid prototyping methods should be of great benefit because they afford integration of additional networked resources, without significant increase in overall system complexity. This ought to give a particular advantage over similar test-bed systems that are not built in quite such a flexible manner [31,32,33]. In practice, it has proved very difficult to persuade medics to code HTML forms to describe their own specialist reports. Nevertheless, we are still able to use such forms through a process of consultation and refinement, whereby medics check the forms that are being compiled by our developers. It seems that most medics are so fully occupied with their traditional work that they are not able to devote time to coding how they do it.

## Discussion

As presented here, our InfoDOM prototype implements a rich, but entirely client-side, EPR system. On the server side, we have been satisfied to transmit and receive XML documents that get stored on the server file-system, just as a typical website deals with plain HTML files. Any practical EPR services, however, must consider more sophisticated server-side solutions that provide: confidentiality on the server, full auditing of the changes made to patient data over time, and the management of transactions when two or more users work simultaneously on the same patient. Some of these features are now becoming available as major database vendors, such as Oracle and Microsoft, incorporate XML processing into their products.

In theory, the tree structure of XML ought to afford an even more finely-grained control over the life of a document than traditional file-locking or database table-locking can do. In particular, problems arising due to the asynchronous nature of web connectivity ought to be ameliorated. For example, conflict resolution done at a tree-branch level would mean that medics working on different parts of the same document ought not to unduly hamper each other's access. We are currently developing a server-side version-control system based on these ideas.

Once an EPR server has established that two medics want to access the same part of the same patient's document, it might be appropriate to establish a peer-to-peer connection between them so that they become aware of each other's work and resolve conflicts before submission to the database. This would constitute a form of instant-messaging - cf, ICQ (I Seek You), AIM (AOL Instant Messenger) - whereby sections of the XML document are exchanged between participating clients. A network-aware JavaBean component connected on the existing InfoBus could be developed to fulfill this purpose.

It is also important that any new EPR server integrates well with existing information systems within the target healthcare institution. An immediate example is that our EPR server ought to able to appropriately handle notification from the PACS of new arrivals of imaging exams. Anagraphic databases and exam-booking systems provide other examples. We plan to use the SOAP (Simple Object Access Protocol) protocol [34] to specify server-to-server calls for information. SOAP protocol has the benefit that it works entirely in XML and runs on top of the standard web protocol. This means that it suffers none of the network accessibility problems that adversely affect CORBA/IIOP, Java/RMI and DCOM approaches. We are currently modeling an existing radiological workflow process using distributed servers that communicate by SOAP.

A problem we have not addressed here is that of the sheer volume of data that image exams contain. What can be done to serve image exams to users that do not have a broadband connection? For example, a consultant might save time by being able to see a particular aspect of an exam while off-site, or working from home. To this end, we are considering possible means for caching on the PACS server a variety of reduced representations of imaging exams, in such a way that the client could prioritize at a distance the order of arrival of the information, so that the medic can see the images in the order that they are needed. Such smart image-delivery mechanisms could be based on image-segmentation algorithms, wavelet compression, or simple tessellation techniques. A key issue here is to understand the human perceptual interface needed to support the medic at a distance.

## Abbreviations

CORBA: Common Object Request Broker Architecture
CSS: Cascading Style Sheet
DCOM: Distributed Component Object Model
DICOM: Digital Imaging and Communications in Medicine
DOM: Document Object Model
EDI: Electronic Data Interchange
EPR: Electronic Patient Record
GP: General Practioner
HTML: HyperText Markup Language
IIOP: Internet Inter-ORB Protocol
OO: Object Oriented
PACS: Picture Archiving and Communications System
RMI: Remote Method Invocation
SGML: Standard Generalized Markup Language
SOAP: Simple Object Access Protocol
SSL: Secure Socket Layer
SVG: Scalable Vector Graphics
XML: eXtensible Markup Language
XSL: eXtensible Style Language
